# Soluble Axoplasm Enriched from Injured CNS Axons Reveals the Early Modulation of the Actin Cytoskeleton

**DOI:** 10.1371/journal.pone.0047552

**Published:** 2012-10-24

**Authors:** Patrick Garland, Lucy J. Broom, Shmma Quraishe, Paul D. Dalton, Paul Skipp, Tracey A. Newman, V. Hugh Perry

**Affiliations:** 1 Centre for Biological Sciences, University of Southampton, Southampton General Hospital, Southampton, United Kingdom; 2 Clinical Neurosciences, Life Sciences Building, University of Southampton, Southampton, United Kingdom; 3 Med-X Research Institute, Shanghai Jiao Tong University, Shanghai, China; 4 Centre for Biological Sciences, Life Sciences Building, University of Southampton, Highfield Campus, Southampton, United Kingdom; Nathan Kline Institute and New York University School of Medicine, United States of America

## Abstract

Axon injury and degeneration is a common consequence of diverse neurological conditions including multiple sclerosis, traumatic brain injury and spinal cord injury. The molecular events underlying axon degeneration are poorly understood. We have developed a novel method to enrich for axoplasm from rodent optic nerve and characterised the early events in Wallerian degeneration using an unbiased proteomics screen. Our detergent-free method draws axoplasm into a dehydrated hydrogel of the polymer poly(2-hydroxyethyl methacrylate), which is then recovered using centrifugation. This technique is able to recover axonal proteins and significantly deplete glial contamination as confirmed by immunoblotting. We have used iTRAQ to compare axoplasm-enriched samples from naïve vs injured optic nerves, which has revealed a pronounced modulation of proteins associated with the actin cytoskeleton. To confirm the modulation of the actin cytoskeleton in injured axons we focused on the RhoA pathway. Western blotting revealed an augmentation of RhoA and phosphorylated cofilin in axoplasm-enriched samples from injured optic nerve. To investigate the localisation of these components of the RhoA pathway in injured axons we transected axons of primary hippocampal neurons *in vitro*. We observed an early modulation of filamentous actin with a concomitant redistribution of phosphorylated cofilin in injured axons. At later time-points, RhoA is found to accumulate in axonal swellings and also colocalises with filamentous actin. The actin cytoskeleton is a known sensor of cell viability across multiple eukaryotes, and our results suggest a similar role for the actin cytoskeleton following axon injury. In agreement with other reports, our data also highlights the role of the RhoA pathway in axon degeneration. These findings highlight a previously unexplored area of axon biology, which may open novel avenues to prevent axon degeneration. Our method for isolating CNS axoplasm also represents a new tool to study axon biology.

## Introduction

Axon injury and degeneration is a common feature of diverse acute and chronic neurological conditions. Following transection, axons retain their structural integrity and are capable of conducting action potentials for a species and tissue specific period of time [1]. However, following this lag phase the injured axons undergo a rapid and dramatic structural reorganisation, which involves blebbing, fragmentation and eventual clearance via phagocytosis [2]. These events have been termed Wallerian degeneration following Augustus Waller’s original transection experiments [3].

Although Wallerian degeneration has been observed across species, the discovery of mice expressing the ‘Wallerian degeneration slow’ (Wld^s^) phenotype has promoted the idea that axon degeneration is a controlled process functionally similar to apoptosis [2], [4]. In the peripheral nervous system of Wld^s^ mice, transected axons of the sciatic nerve survive for over two weeks compared to the approximately 1.5 days of wild-type mice [4]. A similar period of extended survival has also been observed for transected axons in the CNS of Wld^s^ mice [5]. The Wld^s^ protein, and various derivatives of it, has been used to investigate Wallerian degeneration in rodents and other model systems, with the consistent finding that it is protective across species [1].

Many standard experimental techniques, and in particular the use of unbiased screens, have been unavailable to researchers in the field of mammalian axon biology due to the technical limitations of isolating CNS axons from their associated glia, myelinating oligodendrocytes and astrocytes. A variety of approaches have been employed to circumvent this problem. For example, the size and accessibility of various non-mammalian nervous systems have been used to isolate axoplasm, with the squid giant axon and other invertebrate systems being used for some years [6]–[9]. To investigate the biology of mammalian axons, *in vitro* systems have also been used that compartmentalise neuronal processes from their cell bodies [10]–[12]. Recently, a novel technique to isolate axonal proteins from rat sciatic nerve has been described and used to investigate the proteome of injured PNS axons [13], [14].

In order to investigate the proteome of degenerating CNS axons we have developed a novel technique to generate axoplasm-enriched samples from rodent optic nerve. This method employs the use of the dehydrated hydrogel, pHEMA (poly(2-hydroxyethyl methacrylate)) to draw axoplasm from rodent optic nerve. By comparing axoplasm-enriched samples from naïve and injured optic nerve, we have revealed a pronounced modulation of proteins involved in the actin cytoskeleton at time-points that would be expected to occur prior to the large structural changes underlying Wallerian degeneration.

## Results

### Detergent-free Recovery of Soluble Axonal Proteins from Rodent Optic Nerve

**Figure 1:**
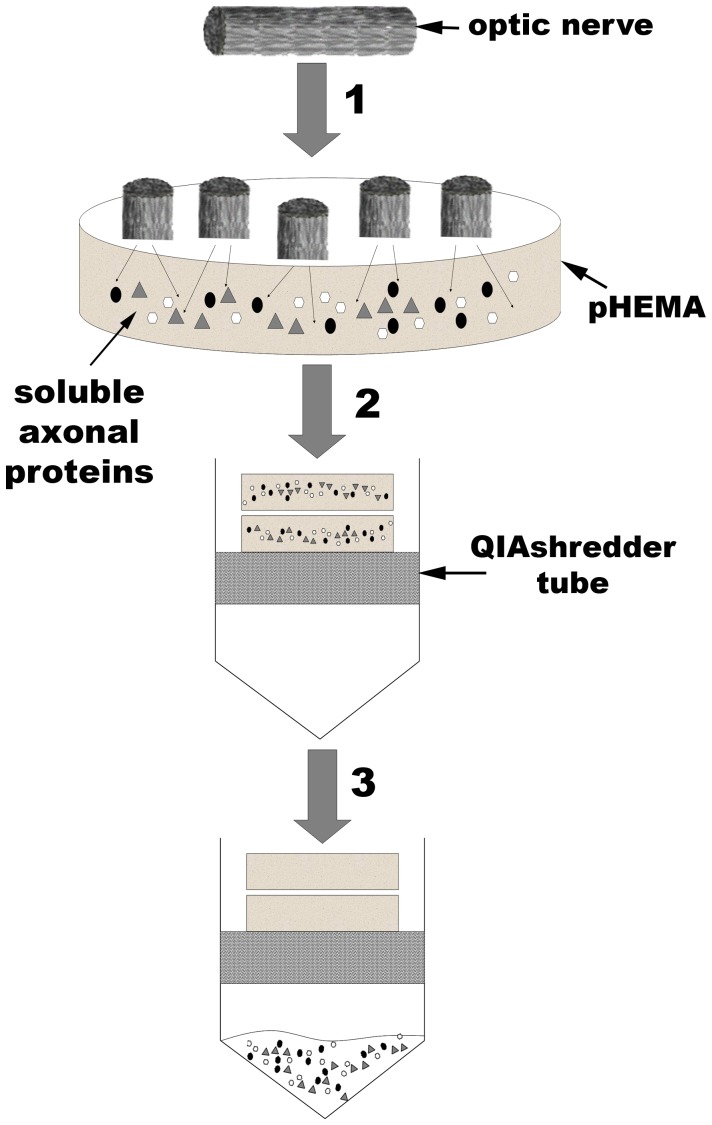
Generation of axoplasm-enriched optic nerve samples using a detergent-free method. (1) Dissected optic nerve is cut into ≈1 mm segments and positioned so that axon orientation is at 90 degrees to the pHEMA disk; optic nerve segments are incubated on the pHEMA disks for 1 hr at 4C. (2) Tissue is removed and two pHEMA disks are placed into a QIAshredder tube, overlaid with 80 ul of axoplasm buffer (0.5 M TEAB + protease inhibitor) and incubated at 4C for 10 min. Tubes are then spun at 12,000 g for 10 min, followed by the addition of a further 50 ul of axoplasm buffer, a 5 min incubation at 4C and a further spin at 12,000 g. (3) Axoplasm-enriched samples are collected and placed at −20C for storage.

**Figure 2:**
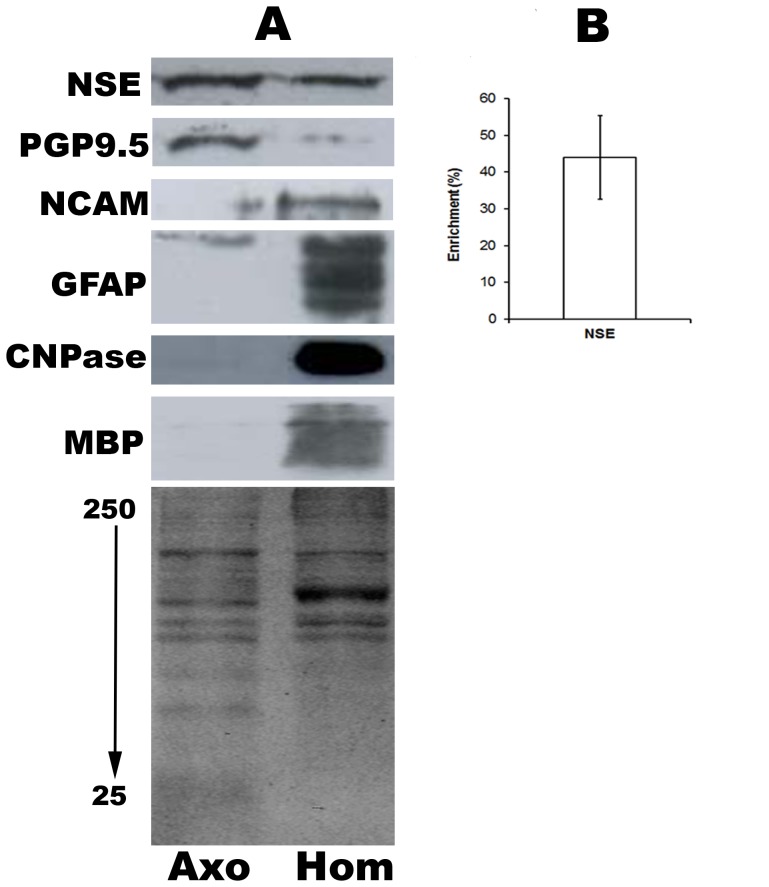
Detergent-free recovery of soluble axonal proteins. Optic nerve homogenates and axoplasm-enriched samples were separated using SDS-PAGE and coomassie stained, which revealed alternative populations of proteins (A). Immunoblotting was performed for a series of axonal and glial markers. Soluble (GFAP and CNPase) and membrane-bound (MBP) glial markers were significantly depleted in axoplasm samples (A). In contrast, the soluble axonal markers NSE and PGP9.5 were recovered and enriched (A&B). The membrane-bound axonal marker NCAM was not similarly recovered (A). Images are representative of at least 3 separate axoplasm preparations. Densitometric analysis of NSE percentage recovery versus homogenate is presented as ±S.E.M (n = 4).

We have developed a detergent-free method for recovering and enriching axoplasm from mammalian optic nerve. Axoplasm is drawn into the pores of the super-absorbent hydrogel, pHEMA and retrieved by centrifugation (Fig. 1). This polymer is produced using cheap and readily accessible reagents, can be modified to suit various applications and has biocompatible properties that has promoted its use in tissue repair, including spinal cord injury [15]. Our method is able to significantly reduce contamination from glial components as well as recover, and enrich for, axonal proteins (Fig. 2).

Coomassie staining of axoplasm-enriched and whole optic nerve samples revealed separate populations of proteins (Fig. 2). The protein content of samples enriched for axoplasm was compared to whole optic nerve homogenate using a series of markers. Western blots for each marker and densitometry analysis, where appropriate, are presented (Fig. 2A&B). We chose 2′, 3′-cyclic nucleotide 3′- phosphodiesterase (CNPase), as a soluble oligodendrocyte marker and myelin basic protein (MBP), as a major constituent of the myelin sheath (Fig. 2A). As can be seen these oligodendrocyte markers were almost completely removed from the axoplasm sample. A similar pattern was also observed for the cytoplasmic astrocyte marker GFAP (Fig. 2A). We assessed the recovery of soluble axonal proteins using antibodies for ubiquitin C terminal hydroxylase (UCH-1/PGP9.5) and neuron specific enolase (NSE). PGP9.5 was highly enriched and NSE was also enriched by over 40% compared to optic nerve homogenate (Fig. 2A&B). In contrast, the membrane-bound marker of optic nerve axons, neural cell adhesion molecule (NCAM), was not readily recovered by this protocol (Fig. 2A).

Others methods employed to isolate axonal material have found contamination of samples with blood components [14]. To investigate whether our samples are similarly contaminated we performed immunoblotting for rat IgG (Fig. S2); we only observed evidence for rat IgG in unperfused animals and not in animals perfused prior to sample isolation. All samples for this study were from perfused animals, and previous work from our lab has established that the blood-brain barrier in optic nerve distal to a crush injury remains intact [16].

### Proteomic Analysis of Axoplasm at 24 and 48 hours after an Optic Nerve Crush

We performed two 4-plex iTRAQ experiments, each of which compared axoplasm from two control and two injured optic nerves. Injured optic nerves were collected at 24 hours after injury for experiment 1 and at 48 hours after injury for experiment 2 the latter time point immediately prior to the onset of degeneration of the majority of optic nerve fibres in rat [17]. This data is deposited on the PRIDE (www.ebi.ac.uk/pride) database under accession numbers 20195 & 20196. Using ProteinLynx Globalserver 2.2, our defined search parameters (Methods section) identified ∼300 proteins in each experiment. Proteins were identified from a wide spectrum of functional groups including; metabolic enzymes, transport proteins, signalling intermediates, structural proteins and proteins involved in translation and protein folding (Fig. S1). Protein changes of magnitude 1.5-fold or more were considered for further investigation. Cytoskeletal proteins, especially those related to the actin cytoskeleton, showed the highest frequency of expression changes after injury. The 27 proteins related to the actin cytoskeleton that showed altered expression at 24 and 48 hours after injury are listed in Table 1.

**Table 1:** Actin cytoskeleton associated proteins changed in injured rat optic nerve compared to naive. All fold changes were p<0.05 significance using a student’s T-test.

UniProt ID	Protein	Function	Fold change
**24hrs post-crush**			
F1MAP4	MEGF10	Guidance signalling	+4.88
Q63170.2	Dynein, heavy chain 7	MT motor protein	+3.15
Q5Y9B8	GAP1	Signalling intermediate	+1.51
F1LQ75	Myosin 1G	Actin motor protein	−2.02
Q9Z2L0	Voltage dependent anion channel 1	Mitochondrial metabolite channel, regulated by actin	−1.89
G8CYZ7	Engulfment & cell motility 1 (ELMO)	Cytoskeletal dynamics via DOCK	−1.67
Q8R5H2	Cadherin 10	Cell-cell adhesion	−1.51
**48hrs post-crush**			
P70566	Tropomodulin 2	Blocks actin depolymerization	+2.48
D3ZFK8	Farp2	Rho GEF	+1.68
Q91XN7	Tropomyosin 1	Stabilizes actin filaments	+1.45
Q91XN5	Prominin 1	Membrane protrusions	−9
Q62951	CRMP3	Signal transduction from Semaphorins	−8.87
D3ZCU8	RAB 33A	Intracellular vesicle trafficking	−5.17
O89046	Coronin 1b	F-actin cross-linking	−4.65
F1LS52	ROBO2	Receptor for slit, axonal guidance	−3.9
P48037	Annexin VI	Ca-responsive actin binding	−3.84
O35889	Afadin	Adherens junctions. Profilin interaction	−3.74
P48675	Desmin	Intermediate filament	−3.35
O35763	Moesin	Connects cytoskeleton to membrane	−2.65
Q8CFN2	Cdc42	Rho GTPase	−2.07
Q9WU34	Septin 3	Ras GTPase	−1.98
P0C6P5	p190-RhoGEF	Rho A activator	−1.93

### The RhoA Pathway is Modulated in Injured Optic Nerve

The iTRAQ data showed altered levels of actin stabilizing proteins (tropomodulin 2 and tropomyosin 1), structural connectors (coronin 1b) and upstream signalling proteins (Rho GTP Exchange Factors (RhoGEFs) and Rho GTPases including Farp 1 and synapse defective 1. We therefore investigated whether changes to proteins involved in actin filament stability might occur following injury. We chose to focus on the modulation and activation of the RhoA pathway, which is known to signal to the actin cytoskeleton [18].

**Figure 3:**
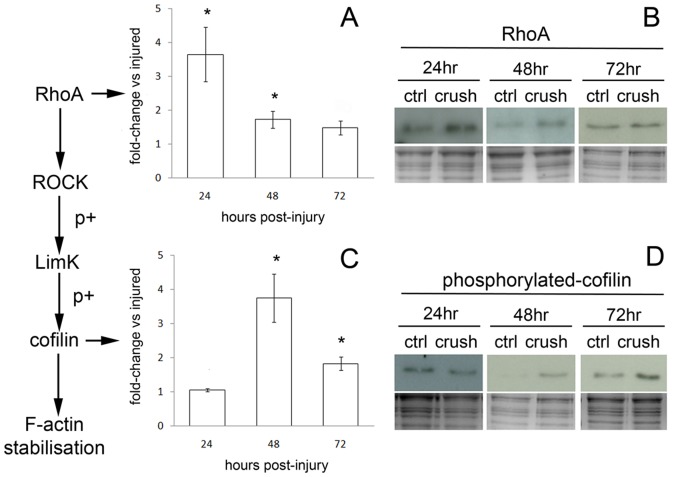
RhoA and phosphorylated cofilin expression are modulated in axoplasm-enriched samples from injured optic nerve. Injured optic nerves and control contralateral nerves were collected at 24, 48 and 72 hrs and axoplasm-enriched samples generated. Immunoblots for RhoA and phosphorylated cofilin are shown, with coomassie stained gels to determine loading (B&D). Quantification is presented as fold-change vs injured and represent 3 independent experiments ±SEM (A&C); one-way ANOVA plus Tukey post hoc test was used to assess significance, and where indicated (*) equals p<0.05. Immunoblotting for RhoA protein revealed a significant increase at 24 hrs, which persisted up to 48 hrs following injury (A&B). Following the increase in RhoA we observed a lag of 24 hrs before a significant increase in phosphorylated cofilin was detected that persisted until 72 hrs post-injury (C&D).

We collected axoplasm-enriched samples from control nerves and at 24, 48 & 72 hr time-points following optic nerve injury for immunoblotting analysis. Protein loading was assessed by running coomassie gels in parallel to those for western blotting. Immunoblotting for total RhoA protein revealed a significant increase at 24 & 48 hr (Fig. 3A&B).

RhoA activation is known to lead to phosphorylation of cofilin via the kinases LIM and ROCK (3). Following phosphorylation, cofilin loses its capacity to sever filamentous actin (F-actin), which can subsequently increase the stability of F-actin within cells. We therefore hypothesised that the observed increase in RhoA expression could result in an increase in cofilin phosphorylation. Immunoblots for phosphorylated cofilin revealed an increase at 48 hrs that persisted until 72 hrs post-injury (Fig. 3C&D). Taken together, these data reveal a modulation and activation of the RhoA pathway in axoplasm-enriched samples isolated from injured optic nerve.

### The RhoA Pathway is Modulated in Transected Processes *in vitro*

To confirm the modulation of the RhoA pathway in injured axons we used the transection of the processes of cultured primary neurons as our model. In particular, we used the method developed by Fath et al (2009) to culture hippocampal neurons at low density and therefore allow the transection of isolated axons, which we analysed using confocal microscopy. Using this system axons were readily identifiable by their distance from the soma, morphology and positive staining for tau as revealed by the monoclonal antibody PHF-1 [19], [20].

**Figure 4:**
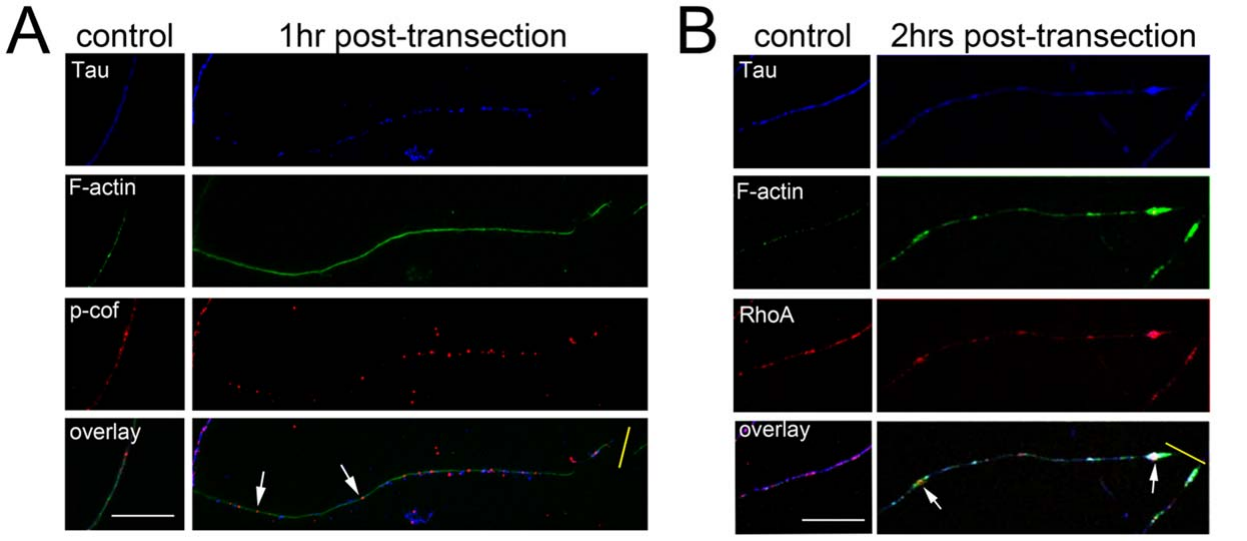
RhoA, phosphorylated cofilin and filamentous actin (F-actin) are modulated in injured axons *in vitro*. Primary hippocampal neurons (14DIV) were cultured according to the method of Fath et al (2009) and stained for Tau, RhoA, p-cofilin, and F-actin at 1–2 hrs following transection of their neurites. Uninjured axons revealed amorphous puncta for RhoA, p-cofilin and F-actin that were distributed stochastically with a variable degree of co-localisation (A&B control panels). One hour following transection (yellow bar) F-actin staining appeared smooth and more intense with a concomitant redistribution of p-cofilin into widely-spaced circular puncta as indicated with arrows (A). After 2 hrs following transection, RhoA was observed to be concentrated in F-actin rich structures (B); in particular, large RhoA-positive swellings were observed (arrows), which also occasionally presented as enriched in tau (B). Scale bar = 10 µM.

We investigated the localisation of RhoA and phosphorylated-cofilin (Fig. 4A&B). Activation of the RhoA pathway can promote the formation of F-actin [18], which we assessed using the fungal toxin phalloidin.

In uninjured tau-positive axons we observed amorphous puncta positive for phosphorylated-cofilin and filamentous actin that were distributed stochastically throughout the processes (Fig. 4A). However, 1 hr following transection a subset of axons exhibited a dramatic change in their staining for these markers: we observed that staining for filamentous actin became smooth and extended throughout the process and was associated with large circular puncta positive for phosphorylated cofilin, which were widely spaced throughout the transected axons (Fig. 4A).

Like the staining for phosphorylated cofilin, the staining for RhoA presented as amorphous puncta distributed randomly throughout tau-positive axons, which did not exhibit an absolute association with filamentous actin (Fig. 4B). In contrast, 2 hrs following transection tau-positive axons exhibited large RhoA and F-actin positive swellings distal to the transection site (Fig. 4B) but also present proximally. These observations confirm that modulation of RhoA, cofilin & F-actin is an early molecular event following injury to axons.

## Discussion

The field of axon biology requires *in vitro* and *in vivo* methods that provide simple and effective approaches to isolate axonal material. Our detergent-free technique significantly depletes glial contamination while recovering soluble axonal proteins. Proteomic analysis of axoplasm-enriched samples from injured vs uninjured optic nerve has revealed a pronounced modulation of proteins regulating the actin cytoskeleton. Importantly these samples were harvested at intervals prior to the loss of axon conduction of the majority of medium and fine axons of the optic nerve and prior to signs of axon disintegration as assessed with the electron microscope [17]. The proteomic changes thus describe some of the earliest events that follow axon injury.

As an initial confirmation of our proteomics screen we focused on the RhoA pathway. Analysis of protein abundance by western blotting has confirmed an increase in RhoA protein in axoplasm-enriched samples distal to an optic nerve crush. Using immunocytochemistry on primary hippocampal neurons we have observed the possible translation and/or redistribution of RhoA into F-actin rich structures, in particular swellings. Swellings distal and proximal to axonal transections have been observed *in vivo*, and RhoA protein has been shown to accumulate in swollen neurites in vivo following spinal cord injury [21]–[23]. Our data therefore confirm and extend these observations. Interestingly, we also observed the occasional enrichment of tau at these swellings, which further supports the observation that the axonal cytoskeleton is reorganised during axon degeneration. Analysis by western blotting of axoplasm-enriched samples from crushed optic nerve also revealed an increase in phosphorylated cofilin. *In vitro*, this increased phosphorylation of cofilin presented as a change in its distribution along transected axons, which was accompanied by a redistribution of filamentous actin.

The actin cytoskeleton forms a matrix that is tethered to the plasma membrane and is organised and regulated by many actin binding proteins (ABPs); it organises the time and place of multiple cellular processes, plays an essential role in facilitating the morphological changes that underlie growth cone and lamellipodia motility, and detaches from the cell membrane to promote blebbing [24], [25]. Wallerian degeneration involves the blebbing of the axolemma, which is subsequently followed by fragmentation of the axon [26]. Our proteomic analysis of degenerating axons has shown a pronounced reduction of ABPs, which would be expected to effect the integrity of the actin cytoskeleton. This post-injury reduction of ABPs may therefore be an initiating event in axolemma blebbing, and subsequently the morphological changes that are necessary for axon fragmentation. Genetic disruption of the calcium-dependent protease calpain reduces blebbing in MEF cells, as well as modulating a set of ABPs similar to those we have identified [27]; this therefore suggests a regulatory connection between ABPs and calpain, which is itself a known regulator of Wallerian degeneration [28].

ABPs are necessary for regulating the pool of dynamic actin [24]. Pharmacologically manipulating the transfer between globular and filamentous actin has revealed an essential role for the actin cytoskeleton in sensing cellular viability across multiple eukaryotes, and subsequently promoting apoptosis when viability is compromised [24], [29], [30]. In particular, inhibiting the formation of F-actin can promote cytochrome c release from mitochondria, and certain ABPs regulate apoptosis via altering mitochondrial function [24], [31]. The recent report that mutations in the actin-binding protein, profilin, underlie familiar cases of amyotrophic lateral sclerosis further illustrates the requirement for a functional actin cytoskeleton in axon viability [32].

We focused on the RhoA pathway as a major modulator of the actin cytoskeleton. There have been a number of reports suggesting the activation of the RhoA pathway in axons following spinal cord injury [33], [34]. However, these reports suggest that extrinsic signals initiate the activation of the RhoA pathway; for example via components of myelin. In contrast, our data would suggest that axons have the intrinsic capacity to activate the RhoA pathway following physical injury. For example, calcium influx is a known regulator of Wallerian degeneration in transected axons and RhoA activation occurs in HUVEC cells following calcium influx [35], [36]. Alternatively, an integrin, but not calpain, dependent activation of Rho-kinase has been reported to result from mechanical injury of axons *in vitro*, and the transcriptome of organotypic hippocampal slices subjected to a stretch injury has identified the upregulation of RhoA [37], [38].

A role for modulation of actin and actin binding proteins has been described in diseases where neurodegeneration results in axon degeneration. For example, axonal inclusions consisting of cofilin-actin ‘rods’ have been observed in cases of Alzheimer’s disease [39].

We have presented a novel method to isolate axoplasm from CNS tissue, which can facilitate the study of axon biology. Proteomic analysis of axoplasm-enriched samples from injured nerves has identified the actin cytoskeleton as a novel point of regulation in axon degeneration and supports data across multiple eukaryotes showing this component of the cytoskeleton as essential for sensing cell viability.

## Materials and Methods

### Ethics Statement

All animal procedures were carried out under the Home Office Animal Act (UK) 1986 under the project licence PPL70/5972.

### Surgical Procedures and Tissue Collection

Adult male Wistar rats (Harlan, UK) were anesthetised with isofluorane. The left optic nerve was crushed intra-orbitally for 10 seconds using curved forceps and the animal was allowed to recover. Experimental and control animals were terminally anesthetised with sodium pentobarbital (200 mg/kg, i.p.) and perfused with 1% heparin/0.9 M saline. Optic nerve tissue from control (uninjured) and crushed optic nerves were collected from the site of injury to the optic chiasm at time points of 24 and 48 hrs after injury and snap-frozen.

### Preparation of Axoplasm-enriched Protein Samples

For axoplasm enrichment, sheets of poly(2-hydroxyethyl methacrylate) (pHEMA) were cast to a thickness of 1.5 mm at 4°C, overnight, with polymerization of the monomer, 2-hydroxyethyl methacrylate (HEMA, Sigma-Aldrich), in excess water (3.955 g dH2O, 932 µl HEMA). Polymerization was initiated using ammonium persulphate (0.5 wt. %, Sigma-Aldrich) and *N*, *N*, *N*′, *N*′-tetramethylethylenediamine (0.2 wt. %, Sigma-Aldrich). The hydrogel was cut into small discs using a sterile borer and freeze-dried overnight to produce a highly absorbent xerogel. Axoplasm enrichment from a single rat optic nerve was performed by the protocol outlined in Figure 1. The protein concentration of eluted soluble proteins, axoplasm, was determined using the Bicinchoninic acid protein assay (Perbio Science). Samples were stored at –20C prior to use.

### iTRAQ

For iTRAQ experiments, axoplasm was prepared from two control and two crushed optic nerves and labelled with iTRAQ reagents, according to the manufacturers protocol (Applied Biosystems). Control axoplasm peptides were labelled with iTRAQ reagents 114 and 115, while crush axoplasm peptides were labelled with reagents 116 and 117. The four samples were combined and lyophilised *in vacuo*, then separated by strong cation exchange (SCX) chromatography on a Dionex Ultimate nano-LC system, using a Polysulfoethyl A column (2.1 mm i.d.×150 mm, 5 µm, 300Å, Phenomenex). The sample was loaded and washed using 25% (v/v) acetonitrile, 10 mM phosphoric acid for 20 min, at 200 µl/min. Peptides were eluted with a linear gradient of 0–500 mM KCl in 25% (v/v) acetonitrile, 10 mM phosphoric acid, at 200 µl/min and fractions collected at 1 min intervals. Fractions were lyophilised *in vacuo,* then re-constituted in 50 µl of MilliQ® - water and separated by nano - RP-LC using a PepMap C_18_ guard column (5 mm×300 µm i.d. Dionex) and a C_18_ reverse phase PepMap column (150 mm×75 µm i.d, Dionex). The resulting peptides were electrosprayed into a Q-tof mass spectrometer (Waters Ltd). Two independent experiments were performed using tissue collected at 24 and 48 hours after injury.

### MS/MS and Data Processing

All data were acquired using a Q-tof Global Ultima (Waters Ltd) fitted with a nanoLockspray. Mass spectra were automatically processed and searched against the Swissprot Rat database (March 2006) using ProteinLynx Global Server 2.2 (Waters Ltd). A maximum of one missed cleavage for tryptic digestion and fixed modifications for methyl methane-thiosulfonation of cysteine and the N-terminus and lysine side chains using the 4-plex iTRAQ label were allowed (Applied Biosystems, Warrington, UK). Variable modification for the oxidation of methionine and iTRAQ modification of tyrosine were also allowed. Precursor ion and sequence ion mass tolerances were set at 100 ppm and 0.1 Da respectively. All spectra were manually checked, to ensure protein identifications were based on accurate assignment of MS/MS fragment ions. Quantitative data was extracted using an iTRAQ parser and average iTRAQ reporter ion intensities for control and crush samples were compared.

### Western Blotting

To compare axoplasm samples to optic nerve homogenates, optic nerves were homogenised in 1X RIPA buffer (10X RIPA: 0.5 M Tris, 1.5 M NaCl, 2.5% sodium deoxycholate, 10% NP40). As described above, axoplasm was isolated using 0.5 M triethylammonium bicarbonate (TEAB) supplemented with protease inhibitor (Complete, Roche). Therefore, to ensure both axoplasm and homogenate samples were in equivalent buffers, the RIPA used for homogenisation was made in 0.5 M TEAB and supplemented with protease inhibitor, and axoplasm samples were taken to 1X RIPA using the 10X stock. The protein content of samples in equivalent buffers was then measured using the bicinchoninic acid protein assay (Perbio Science). To visualise the protein content of samples and check protein loading, samples were separated using SDS-PAGE and stained using BioSafe Coomassie (Bio-Rad). Densitometry was performed using Quantity One software (Bio-Rad).

Equivalently loaded samples were separated by SDS-PAGE and transferred to nitrocellulose. Membranes were blocked using tris-buffered saline-0.1% Tween-20 (TBST) containing 3% BSA (blocking buffer). Membranes were then incubated with primary antibodies (Table S1) overnight in blocking buffer. Following 3X washes with TBST, membranes were incubated with HRP-conjugated IgG (Pierce: 32260) in blocking buffer for 1 hr, before developing using ECL (SuperSignal West Pico, Pierce) and Amersham Hyperfilm. When appropriate, dilutions of samples were used to ensure conditions were performed within the linear range of the ECL.

### Primary Neuronal Cultures

Primary neuronal cultures were prepared essentially as described by Fath et al (2009), with some modifications. This method utilizes a ‘support ring’ of cortical cells in the periphery of a culture dish to provide trophic support to a low density culture of hippocampal neurons on a central coverslip. Briefly, p0 C57BL/6 mice were sacrificed and their hippocampal and cortical neurons dissociated using a papain dissociation kit (Worthington). Cortical and hippocampal cells were suspended in growth media (Neurobasal, 1X B27 & 1X GlutaMAX; Invitrogen) and plated at the following densities: 250,000/75 ul cortical cells for the support ring, and 1000/50 ul hippocampal cells per coverslip in 12well plates. After 4 hrs, suspension media was removed and replaced with 1 ml of pre-equilibrated growth media. Every 3–4 days 50% of culture media was replaced with fresh and pre-equilibrated growth media. Cultures were used after 14DIV.

To transect processes a curved blade was held within forceps and allowed to roll over the surface of the coverslip.

### Immunocytochemistry

Culture media was aspirated from wells, which were then washed with phosphate-buffered saline (PBS). Neurons were fixed using room temperature 4% paraformaldehyde for 15 min. Wells were washed X3 5 min with PBS and blocked/permeabilised with blocking buffer (5% normal goat serum, 1% BSA and 0.2% triton X-100 in PBS) for 30 min. Primary antibodies (Table S1) were diluted in blocking buffer and incubated with coverslips overnight at 4C. Coverslips were then washed X3 5 min with PBS before incubation with appropriate fluorophore-conjugated reagents (Alexa488-phalloidin 1/100, anti-rabbit Alexa546 1/500 & anti-mouse Cy5; Invitrogen) for 2 hrs in blocking buffer. Coverslips were washed X3 5 min with PBS, and then mounted using Prolong Gold (Invitrogen). Images were collected using a Leica SP5 confocal microscope (LAS-AF software v2.1.2) using an X100(1.4na) HCX PL APO CS objective. Sequential imaging (AF-488+Cy5/AF546) was performed with photomultiplier gains set to zero signal on negative control samples (i.e. secondary antibody only) to eliminate spectral bleed through between fluorochromes. Background correction was performed on maximum projection images (488+546 channels) and a median filter (radius = 3) passed over the background corrected images to reduce image background.

## Supporting Information

Figure S1 **Functional characterization of protein changes identified after axon injury.** Using Panther software and database searches, proteins were assigned to a particular molecular function. Pie charts illustrate the frequency of protein changes belonging to each group at 24 hours (A) and 48 hours (B) after injury.
(TIF)

Figure S2 **Samples from perfused animals are not contaminated with blood borne components.** Equally loaded samples (5ug/lane; coomassie stain in bottom panel) corresponding to **o**ptic nerve homogenate (H(+p)) and axoplasm-enriched (Axo) from perfused animals were compared to homogenates from unperfused animals (H(-p)) and a 1/10 dilution of rat sera as a positive control. Neither the heavy or light chain of rat IgG was detectable in samples from perfused animals using this technique; in contrast, samples from unperfused animals showed a signal for the heavy chain of IgG.
(TIF)

Table S1 **Antibody list.**
(DOCX)
