# Supplementary figures and images for: Soluble Axoplasm Enriched from Injured CNS Axons Reveals the Early Modulation of the Actin Cytoskeleton
Source: PLoS One. 2012 Oct 24;7(10):e47552. doi: 10.1371/journal.pone.0047552 (PMC3480358; doi:10.1371/journal.pone.0047552)

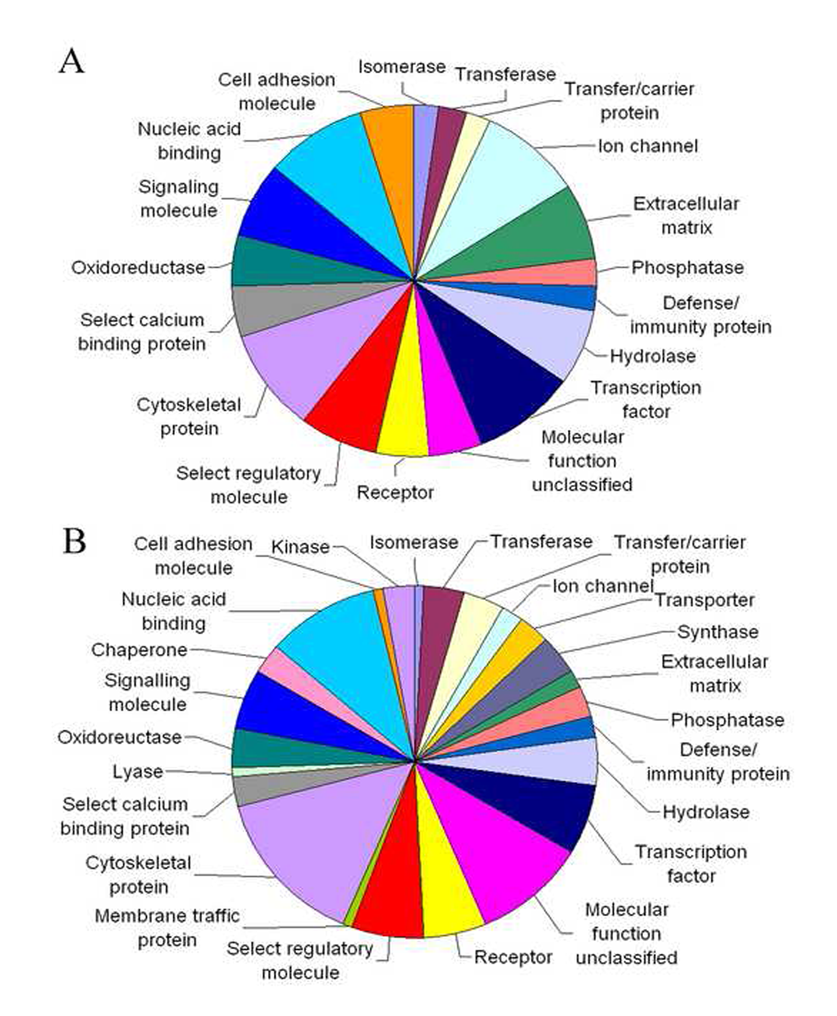

Supplement: Figure S1 — Functional characterization of protein changes identified after axon injury. Using Panther software and database searches, proteins were assigned to a particular molecular function. Pie charts illustrate the frequency of protein changes belonging to each group at 24 hours (A) and 48 hours (B) after injury. (TIF) [file pone.0047552.s001.tif]

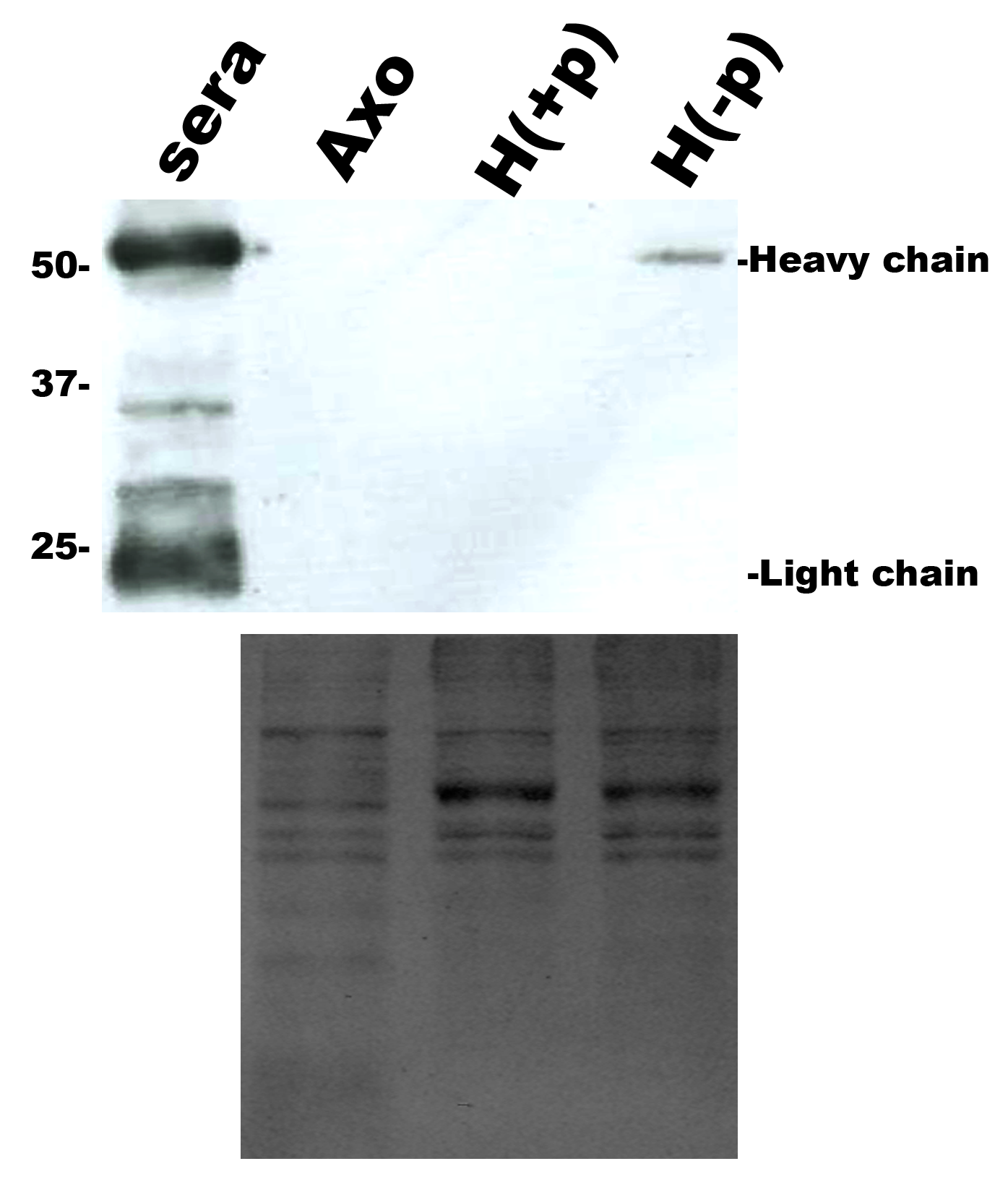

Supplement: Figure S2 — Samples from perfused animals are not contaminated with blood borne components. Equally loaded samples (5ug/lane; coomassie stain in bottom panel) corresponding to optic nerve homogenate (H(+p)) and axoplasm-enriched (Axo) from perfused animals were compared to homogenates from unperfused animals (H(-p)) and a 1/10 dilution of rat sera as a positive control. Neither the heavy or light chain of rat IgG was detectable in samples from perfused animals using this technique; in contrast, samples from unperfused animals showed a signal for the heavy chain of IgG. (TIF) [file pone.0047552.s002.tif]
