# Supplementary material for: Soluble Axoplasm Enriched from Injured CNS Axons Reveals the Early Modulation of the Actin Cytoskeleton
Source: PLoS One. 2012 Oct 24;7(10):e47552. doi: 10.1371/journal.pone.0047552 (PMC3480358; doi:10.1371/journal.pone.0047552)
Supplement: Table S1 — Antibody list. (DOCX) [file pone.0047552.s003.docx]

| **Antibody** | **Product code** | **Supplier** | **Dilution** |
| --- | --- | --- | --- |
| anti-NSE | MAB314 | Millipore | 1/1000 |
| anti-PGP9.5 | ab10404 | Abcam | 1/500 |
| anti-NCAM | AB5032 | Millipore | 1/2000 |
| anti-GFAP | Z0334 | Dako | 1/5000 |
| anti-CNPase | ab6319 | Abcam | 1/1000 |
| anti-MBP | 05-675 | Upstate | 1/5000 |
| anti-RhoA | ab50540 | Abcam | 1/500 |
| anti-phosphorylated cofilin (Ser3) | sc-21867-R | Santa Cruz | 1/500 |
| Anti-Tau (PHF-1 monoclonal) | N/A | P.Davies, The Feinstein Institute for Medical Research, NY. | 1/500 |
| Anti-Rat IgG | BA-4001 | Vector Labs | 1/5000 |

Table S1. Antibody list.
